# Baseline white blood cell count-to-apolipoprotein A1 ratio as a novel predictor of long-term adverse outcomes in patients who underwent percutaneous coronary intervention: a retrospective cohort study

**DOI:** 10.1186/s12944-020-01206-w

**Published:** 2020-03-16

**Authors:** Ying Pan, Jian Zhang, Ting-Ting Wu, Xian-Geng Hou, Yi Yang, Xiang Ma, Yi-Tong Ma, Ying-Ying Zheng, Xiang XIE

**Affiliations:** 1 Department of Cardiology, First Affiliated Hospital of Xinjiang Medical University, Urumqi, 830011 People’s Republic of China; 2 Department of Cardiology, First Affiliated Hospital of Zhengzhou University, Zhengzhou, 450052 People’s Republic of China

**Keywords:** White blood cell count, Apolipoprotein A1, Coronary artery disease, Mortality, Outcomes

## Abstract

**Background:**

Previous studies suggested that baseline white blood cell count and apolipoprotein A1 levels were associated with clinical outcomes in patients with coronary heart disease (CAD) who underwent percutaneous coronary intervention (PCI). However, the ratio of baseline white blood cell count-to-apolipoprotein A1 level (WAR) and CAD after PCI have not been investigated. The present study investigated the effects of baseline WAR on long-term outcomes after PCI in patients with CAD.

**Methods:**

A total of 6050 patients with CAD who underwent PCI were included in the study. Of these, 372 patients were excluded because no baseline white blood cell counts or apolipoprotein A1 (ApoA1) data was available or because of malignancies or other diseases. Finally, 5678 patients were enrolled in the present study and were divided into 3 groups according to WAR value: lower group - WAR< 5.25 (*n* = 1889); median group - 5.25 ≤ WAR≤7.15 (*n* = 1892); and higher group - WAR≥7.15 (*n* = 1897). The primary endpoint was long-term mortality, including all-cause mortality (ACM) and cardiac mortality (CM), after PCI. The average follow-up time was 35.9 ± 22.6 months.

**Results:**

A total of 293 patients developed ACM, including 85 (4.5%) patients in the lower group, 90 (4.8%) patients in the median group, and 118 (6.2%) patients in the higher group. The risk of ACM, cardiac mortality (CM), major adverse cardiovascular and cerebrovascular events (MACCEs), and major adverse cardiovascular events (MACEs) increased 62.6% (hazard risk [HR] =1.626, 95%CI: 1.214–2.179, *P* = 0.001), 45.5% (HR = 1.455, 95%CI: 1.051–2.014, *P* = 0.024), 21.2% (HR = 1.212, 95%CI: 1.011–1.454, *P* = 0.038), and 23.8% (HR = 1.238, 95%CI: 1.025–1.495, *P* = 0.027), respectively, as determined by multivariate Cox regression analyses comparing the patients in the higher group to patients in the lower group. Patients with a WAR≥4.635 had 92.3, 81.3, 58.1 and 58.2% increased risks of ACM, CM, MACCEs and MACEs, respectively, compared to the patients with WAR< 4.635. Every 1 unit increase in WAR was associated with 3.4, 3.2, 2.0 and 2.2% increased risks of ACM, CM, MACCEs and MACEs, respectively, at the 10-year follow-up.

**Conclusion:**

The present study indicated that baseline WAR is a novel and an independent predictor of adverse long-term outcomes in CAD patients who underwent PCI.

## Introduction

A large number of epidemiological and clinical studies have shown that the inflammatory response is closely related to the occurrence and development of coronary artery disease (CAD) [1–4]. Inflammation plays an increasingly important role in major cardiac adverse events (MACE). White blood cell (WBC) count is an inflammatory marker in routine blood tests, and it has a negligible effect on the clinical outcomes of patients with CAD. As early as the 1980s, Schlant et al. observed an increase in WBC count as an indicator of mortality in patients with myocardial infarction [5]. Lao D et al. further demonstrated that leukocytosis is an independent predictor in CAD patients after PCI [6]. A large number of recent studies confirmed that the increase in baseline WBC count is associated with the occurrence of adverse clinical outcomes after PCI in patients with CAD [7–9].

Epidemiological study demonstrated a strong negative correlation between high-density lipoprotein (HDL) levels and atherosclerosis [10]. ApoA-1 is a main protein component of HDL, and it showed a similar negative correlation. A large number of preclinical studies support epidemiological data from animal models of atherosclerosis, which suggest that HDL/apoA-1 intervention reduces plaque size and inflammation [11–15]. Lower HDL levels (< 0.91 mmol/l) doubled patient mortality in patients with PCI compared to patients with higher HDL levels (1.24 to 3.1 mmol/l) [16, 17]. ApoA-1 perfusion reduces endometrial formation and vein grafting after carotid artery injury in mice [18] and enhances the re-endothelialization of endothelial injury sites [19]. Studies directly evaluated the effect of apoA-1 systemic perfusion on stent biocompatibility and found that ApoA-1 was associated with a reduction in-stent restenosis and platelet activation and an increase in endothelialization [20].

Previous study has indicated that elevated serum apoA-1 levels may be associated with decreased levels of high-sensitivity C-reactive protein and decreased WBC counts, which may be inflammatory biomarkers for the onset of CAD [21]. Elevated levels of WBC and decreased levels of apoA-1 may be useful markers of CAD risk. However, there are few reports on the correlation between baseline WBC-to-ApoA1 ratios (WAR) and the occurrence and prognosis of CAD. The present study assessed the predictive effect of baseline WAR on long-term outcomes of CAD patients who underwent PCI.

## Patients and methods

### Study population

This study was a single-center retrospective cohort study that investigated the clinical outcomes and risk factors for patients with CAD after PCI (CORFCHD-PCI). In the CORFCHD-PCI study, we collected clinical, angiographic, short-term and long-term outcome data of CAD patients who underwent PCI in the First Affiliated Hospital of Xinjiang Medical University from January 2008 to December 2016. The details of the design are registered at http://www.chictr.org.cn (ChiCTR-ORC-16010153). In the CORFCHD-PCI study, 6050 CAD patients were recruited. The average follow-up period was 35.9 months, and 102 patients were lost during follow-up. The selected criterion for CAD was at least one coronary artery diameter stenosis ≥70%, as confirmed on coronary angiography. An experienced cardiologist performed PCI. The following exclusion criteria for the study were used: 1) preoperative baseline WBC count or ApoA1 data were not be acquired; 2) systemic disease, malignant tumor, inflammatory disease, acute infectious disease, or severe kidney disease; and 3) taking drugs that affect leukocytes before admission. The present study excluded 372 patients for unavailable WBC or apoA1, acute infections, malignancies, or hepatobiliary disease. Finally, 5678 patients were included the present study. In these 5678 patients, 256 patients have previous CAD history and none of these interventions has coronary artery bypass grafting (CABG) vessel. We divided these patients into 3 groups according to WAR values: lower group - WAR< 5.25 (*n* = 1889); median group - 5.25 ≤ WAR< 7.15 (*n* = 1892); and higher group - WAR≥7.15 (*n* = 1897). WAR was calculated as the ratio of baseline WBC count to ApoA1 from the same blood sample.

### Data collection

We collected general demographic data for patients, including smoking, drinking, past medical history, cardiovascular risk factors, and laboratory-related tests. All laboratory-related tests were performed on the second day after admission after fasting for at least 12 h. Serum concentration of blood urea nitrogen (BUN), creatinine (Cr), uric acid (UA), total cholesterol (TC), triglyceride (TG), glucose (GLU), high-density lipoprotein-C (HDL-C), low-density lipoprotein-C (LDL-C), apolipoprotein A1 (apo-AI), lipid-loading protein B (apo-B) and lipoprotein A (Lp (a)) were measured using equipment for chemical analysis (Dimension AR/AVL Clinical Chemistry System, Newark, NJ, USA) employed by the Clinical Laboratory Department of the First Affiliated Hospital of Xinjiang Medical University.

### Clinical diagnosis

Hypertension was defined as blood pressure ≥ 140/90 mmHg at three different times on the same day or treatment with antihypertensive drugs. The diagnostic criteria for hyperlipidemia were defined according to the “Guidelines for the Prevention and Treatment of Dyslipidemia in Chinese Adults” [22]. The diagnostic criteria for diabetes was a clear history of diabetes, the use of hypoglycemic agents, fasting blood glucose ≥7.1 mmol/L, or two-hour postload glucose ≥11.1 mmol/L.

Coronary angiography, interventional therapy, and postoperative reports were performed by experienced coronary intervention specialists. Patients with CAD who underwent PCI received a loading dose of aspirin and clopidogrel preoperatively, and intravenous heparin anticoagulation was routinely used at the start of PCI.

### Endpoints

The primary endpoint of the study was long-term mortality, including all-cause mortality (ACM) and cardiac mortality (death due to coronary heart disease, cardiogenic shock, or sudden death). Secondary endpoints were major adverse cardiac events (MACEs), which were defined as a combination of cardiac death, recurrent myocardial infarction, and target vessel reconstruction, and major adverse cardiac and cerebrovascular events (MACCEs), which were defined as MACE plus stroke, as described previously [23].

### Follow-up

Specially trained professional staff performed the follow-up. All patients were followed via outpatient, inpatient, and telephone follow-up and with questionnaire surveys. The longest follow-up time was 10 years.

### Statistical analysis

All analyses were performed using SPSS 22.0 for Windows statistical software (SPSS Inc., Chicago, IL, USA). For the subsequent analyses, we divided patients into three groups according to WAR value. Continuous variables are expressed as the means ± standard deviation or medians (25 to 75%), and categorical variables are expressed as a percentage. One-way ANOVA was used to evaluate differences between normally distributed numerical variables, and nonnormally distributed numerical variables were analyzed using the Mann-Whitney U test. The chi-squared test was used to compare categorical variables, and the cumulative incidence of long-term prognosis was analyzed by using Kaplan-Meier analysis. The log-rank test was used to compare groups. To establish the COX model, a univariate model analysis was performed for all predictors, and a significant (*P* < 0.05) variable in the univariate analysis was included in the multivariate Cox model. *P* < 0.05 was considered statistically significant.

## Results

### Baseline data and procedural characteristics

**Fig. 1:**
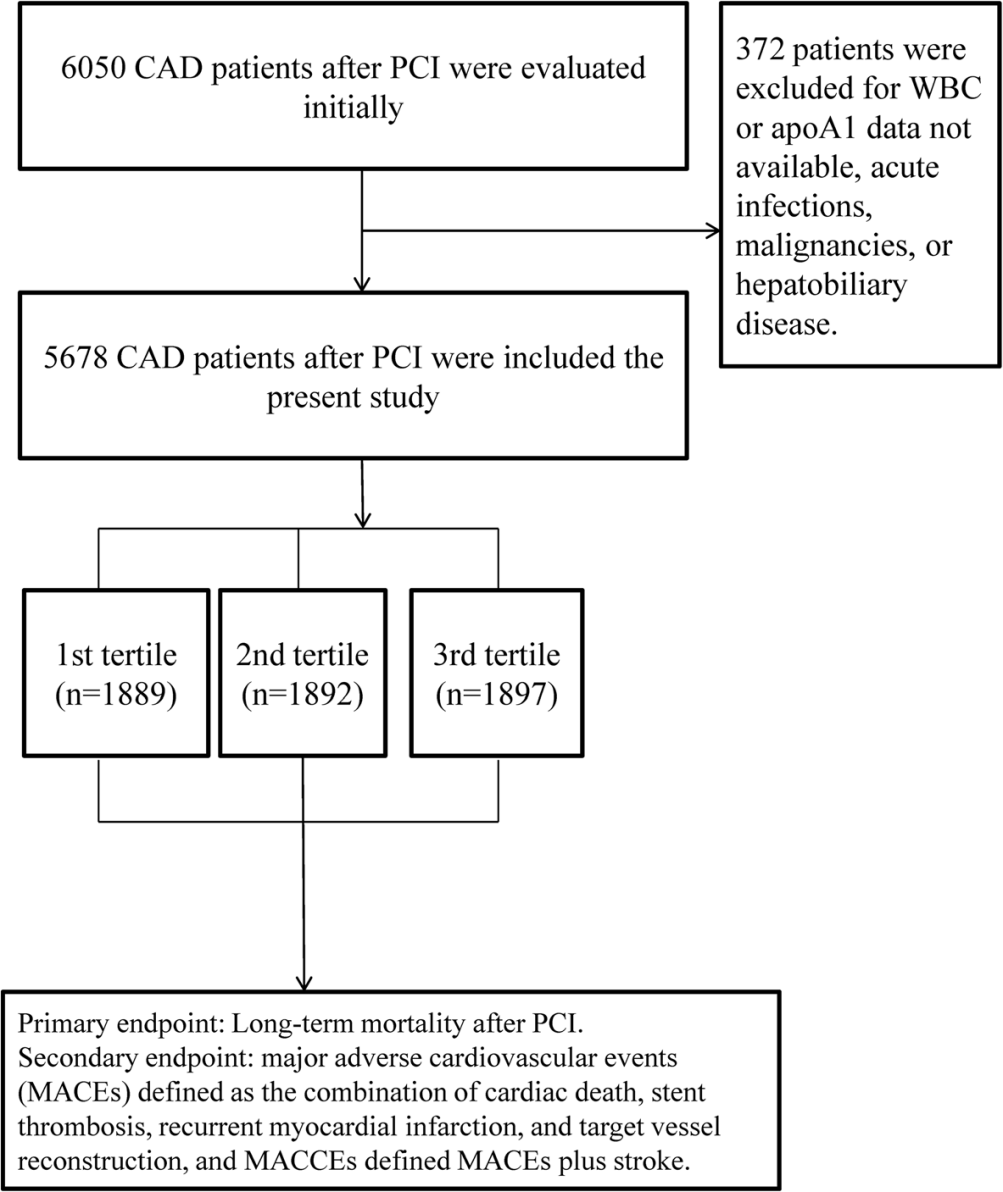
The flow chart of participant inclusion. A total of 6050 CAD patients after PCI were evaluated initially, and 5678 patients were included the present study

As shown in Fig. 1, 6050 patients with CAD who underwent PCI were included in the study. Of these, 372 patients were excluded because of no available baseline white blood cell counts and ApoA1 data, malignancies, and other diseases. Finally, 5678 patients were enrolled in the present study and divided into 3 groups according to WAR: lower group - WAR< 5.25 (*n* = 1889); median group - 5.25 ≤ WAR< 7.15 (*n* = 1892); and higher group - WAR≥7.15 (*n* = 1897). There were significant differences between groups in gender, smoking, alcohol consumption, age, total cholesterol, high-density lipoprotein-C, low-density lipoprotein-C, diastolic blood pressure, creatinine, and uric acid (all *Ps* < 0.05). However, clopidogrel and aspirin treatment, the incidence of diabetes, hypertension, systolic blood pressure, triglycerides, blood urea nitrogen and the procedural characteristics between groups were not significantly different (all *Ps* ≥ 0.05) (Table 1).

**Table 1:** Clinical characteristics in patients stratified by tertiles of WBC-to-ApoA1 ratio (WAR)

Variables	WAR < 5.25	5.25 ≤ WAR< 7.15	WAR > 7.15	*X2*	*P*
	*(n* = 1889)	(*n* = 1892)	(*n* = 1897)		
Clopidogrel, n (%)	544 (29.1)	582 (30.9)	599 (31.8)	3.235	0.198
Aspirin, n (%)	1299 (69.4)	1241 (65.8)	1268 (67.3)	5.478	0.065
Sex, Male, n (%)	1281 (67.8)	1411 (74.6)	1530 (80.7)	81.911	< 0.001
Smorking, n (%)	643 (34.0)	752 (39.7)	885 (46.7)	62.861	< 0.001
Alcohol Drinking, n (%)	459 (24.3)	550 (29.1)	657 (34.6)	48.865	< 0.001
Diabetes, n (%)	457 (24.2)	446 (23.6)	483 (25.5)	1.903	0.386
Hypertension, n (%)	774 (41.0)	826 (43.7)	825 (43.5)	3.492	0.174
Age, years	61.32 ± 10.33	59.68 ± 10.78	57.44 ± 10.90	63.149	< 0.001
SBP, mmHg	126.83 ± 18.80	126.91 ± 18.42	127.48 ± 19.06	0.675	0.509
DBP, mmHg	75.71 ± 11.27	76.23 ± 11.15	76.96 ± 11.51	5.777	0.003
TG, mmol/L	1.86 ± 1.22	1.95 ± 1.36	1.89 ± 1.23	2.796	0.061
TC, mmol/L	4.17 ± 1.13	3.99 ± 1.11	3.73 ± 1.04	76.252	< 0.001
HDL-C, mmol/L	1.14 ± 0.50	1.01 ± 0.40	0.92 ± 0.51	101.364	< 0.001
LDL-C, mmol/L	2.58 ± 0.95	2.48 ± 0.91	2.32 ± 0.86	38.215	< 0.001
Cr, mmol/L	73.97 ± 20.39	76.19 ± 18.97	77.73 ± 21.68	16.248	< 0.001
UA, mmol/L	312.70 ± 87.34	328.98 ± 90.24	327.90 ± 92.32	19.329	< 0.001
BUN, mmol/L	5.49 ± 1.64	5.53 ± 1.62	5.53 ± 1.75	0.366	0.694
GLU, mmol/L	6.36 ± 3.12	6.47 ± 2.92	6.97 ± 3.31	20.61	< 0.001

Note: *SBP* Systolic blood pressure, *DBP* Diastolic blood pressure, *BUN* Blood urea nitrogen, *UA* Uric acid, *Cr* Creatinine, *GLU* Glucose, *TG* Triglyceride, *TC* Total cholesterol, *LDL-C* Low density lipoprotein cholesterol, *HDL-C* High density lipoprotein cholesterol

### Clinical outcomes

**Fig. 2:**
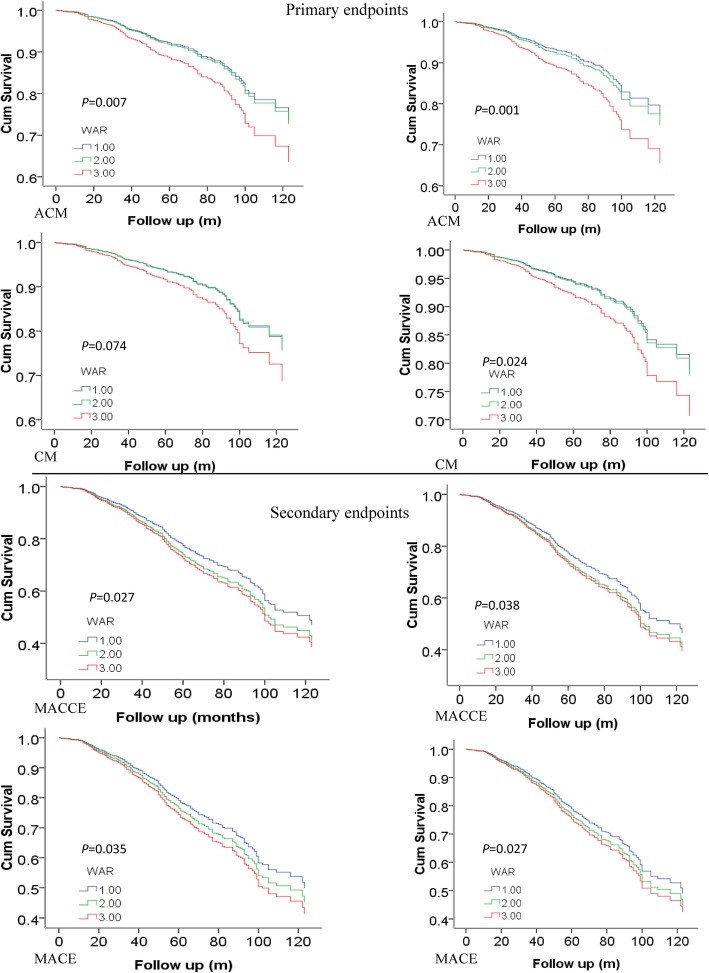
Cumulative Kaplan-Meier estimates of the time to the first adjudicated occurrence of primary endpoints (ACM and CM) and secondary endpoints (MACCEs and MACEs). Left: Univariate analysis; Right: Multivariate. Upper: Primary endpoints; Lower: Secondary endpoints

As shown in Table 2 and Fig. 2, 293 patients developed all-cause mortality (ACM), including 85 (4.5%) patients in the lower group, 90 (4.8%) patients in the median group, and 118 (6.2%) patients in the higher group. The incidence of ACM increased gradually with the increase in WAR value, and the ACM incidence of the higher group was significantly increased than the lower group (HR = 1.484, 95%CI: 1.123–1.962, *P* = 0.007). We also found that the incidence of MACE (HR = 1.267, 95%CI:1.059–1.516, *P* = 0.010) or MACCEs (HR = 1.261, 95%CI: 1.062–1.499, *P* = 0.008) in the higher group was significantly greater compared to the lower group. These differences remained significant after multivariable COX regression analysis (ACM [HR = 1.626, 95%CI: 1.214–2.179, *P* = 0.001], MACCE [HR = 1.212, 95%CI: 1.011–1.454, *P* = 0.038], MACE [HR = 1.238, 95%CI: 1.025–1.495, *P* = 0.027). However, the incidence of CM was not significantly different between groups in univariate analysis (HR = 1.347, 95%CI: 0.989–1.835, *P* = 0.059) but showed significant differences in multivariable analysis (HR = 1.455, 95%CI: 1.051–2.014, *P* = 0.024). The neutrophil-to-lymphocyte ratio (NLR) and monocyte-to-HDL-C ratio (MHR) are independent predictors for outcomes. Therefore, we included these two variables in the multivariable Cox regression analysis. As shown in Tables S1, S2, S3 and S4, WAR remained a strong independent predictor for ACM, CM, MACCEs and MACEs.

**Table 2:** Outcomes comparison between each group

Variables	WAR< 5.25	5.25 ≤ WAR≤7.15	WAR ≥7.15	HR (95%CI)^a^	*P* value	Adjusted HR (95%CI)^a, b^	*P* value
	(*n* = 1889)	(*n* = 1892)	(*n* = 1897)				
ACM, n (%)	85 (4.5)	90 (4.8)	118 (6.2)	1.484 (1.123–1.962)	0.007	1.626 (1.214–2.179)	0.001
CM, n (%)	72 (3.8)	72 (3.8)	91 (4.8)	1.347 (0.989–1.835)	0.059	1.455 (1.051–2.014)	0.024
MACCE, n (%)	240 (12.7)	287 (15.2)	281 (14.8)	1.261 (1.062–1.499)	0.008	1.212 (1.011–1.454)	0.038
MACE, n (%)	221 (11.7)	256 (13.5)	260 (13.7)	1.267 (1.059–1.516)	0.010	1.238 (1.025–1.495)	0.027

Note: *ACM* All-cause mortality, *CM* Cardiac mortality, *MACCE* Major adverse cardiovascular and cerebrovascular events, *MACE* Major adverse cardiovascular events. ^a^HR for comparison of WAR ≥7.15 group with WAR< 5.25 group. ^b^adjustments of age, sex, smoking, drinking, DBP, Cr, UA, GLU, TC, HDL-C and LDL-C

As shown in Table 3, analyses with WAR as a continuous variable showed that every increase of 1 resulted in 3.4, 3.2, 2.0 and 2.2% increased risks of ACM, CM, MACCEs and MACEs at 10-year follow-up, respectively.

**Table 3:** Clinical outcomes and WAR as continuous variable and bisection

Outcomes	HR (95%CI)	*P* values	Adjusted HR (95%CI)	*P* values
ACM				
WAR ≥4.635 vs. < 4.635	1.923 (1.368–2.704)	< 0.001	2.063 (1.460–2.916)	< 0.001
WAR higher by 1	1.034 (1.009–1.060)	0.007	1.034 (1.010–1.059)	0.005
CM				
WAR ≥4.635 vs. < 4.635	1.813 (1.249–2.632)	0.002	1.930 (1.321–2.820)	0.001
WAR higher by 1	1.032 (1.003–1.061)	0.031	1.029 (1.002–1.058)	0.036
MACCEs				
WAR ≥4.635 vs. < 4.635	1.581 (1.307–1.914)	< 0.001	1.586 (1.305–1.928)	< 0.001
WAR higher by 1	1.020 (1.002–1.039)	0.029	1.016 (0.997–1.036)	0.091
MACEs				
WAR ≥4.635 vs. < <4.635	1.582 (1.295–1.931)	< 0.001	1.573 (1.282–1.930)	< 0.001
WAR higher by 1	1.022 (1.003–1.042)	0.022	1.018 (0.998–1.038)	0.080

**Fig. 3:**
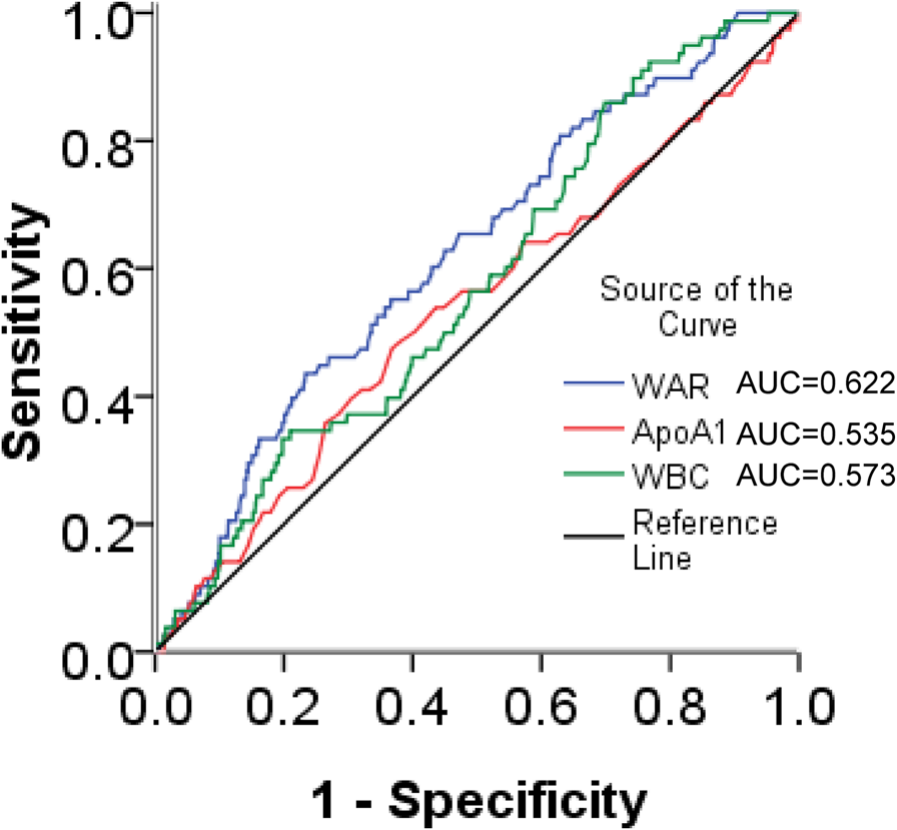
Comparison of areas under the curves between WBC, ApoA1, and WAR

As shown in Fig. 3, the receiver operating characteristic (ROC) curve showed that the area under the curve (AUC) of WAR was the largest (AUC = 0.622) compared to WBC and ApoA1, which suggests that WAR is a stronger predictor for adverse outcomes in CAD patients who underwent PCI than WBC or ApoA1 alone. The ROC curve revealed 4.636 as the optimal cut-off value for WAR. Patients with a WAR ≥4.635 had 92.3, 81.3, 58.1 and 58.2% increased risks of ACM, CM, MACCEs and MACEs, respectively, compared to patients with WAR< 4.635 (Table 3).

## Discussion

The present study suggests that high WAR is significantly associated with long-term prognosis in patients with CAD who underwent PCI. The inclusion of WAR in clinical predictive models may significantly improve the power of prediction in CAD patients after PCI.

Recent studies demonstrated that elevated WBC counts were associated with an increased risk of CAD [22]. Leukocytes are associated with the destruction of arterial plaque stability, and an increased WBC count is an independent risk factor for CAD [24]. A recent study reported that infusion of apoA-1 reduced the neointimal area in the stent in a unique stent-implanted mouse model [20, 25]. Leukocytes are associated with neointimal hyperplasia (NIH) because these cells release inflammatory proteins and growth factors that stimulate smooth muscle cell proliferation [26]. These studies indicate that apoA-1 inhibits smooth muscle cell proliferation, inflammation, and leukocyte recruitment. Therefore, joint analyses of leukocytes and ApoA-1 provide more useful information for the predicting of cardiovascular events in patients with CAD.

The protective effect of ApoA1 on the formation of atherosclerotic lesions was extensively studied in animal models and humans [27, 28]. Yvan-Charvet et al. found that ApoA1 inhibited the degree of leukocytosis to a certain extent, which inhibited the progression of atherosclerosis [29]. The combination of leukocyte subtypes or the combination of leukocyte subtypes with other indicators may be used as a predictor of prognosis in patients with CAD [30]. For example, the neutrophil-to-lymphocyte ratio (NLR) is an independent predictor of long-term cardiovascular outcome after elective PCI [30]. Arisoy A et al. also found that the ratio of monocytes-to-high-density lipoprotein (MHR) was an independent predictor of increased thrombus burden after PCI in patients with STEMI [31]. These studies and findings provide strong evidence to support our current research. Furthermore, previous studies suggested that ApoA1 was associated with cardiovascular events in rheumatoid arthritis patients [32] and CAD patients who underwent PCI [33, 34]. These studies were in line with our result.

Numerous biomarkers and scoring systems are used for prognostic evaluations of patients with CAD. However, some systems are relatively expensive or difficult to apply in clinical practice. The present study is the first to demonstrate the association of elevated WAR with an increased risk of adverse outcomes in CAD patients.

The present study has several limitations. First, as a single-center observational study, unknown confounding factors may have affected the outcome. Second, this study only performed assessments of WAR, and it did not assess the effect of WAR changes. Third, there were 102 patients were loss of follow-up in the study, which might impact on the reality of the result. Forth, we did not compare WAR and well known inflammatory markers in CAD. Finally, the mechanism of action between Apo-A1 and WBC requires further study.

## Conclusion

The present study demonstrated that WAR is independently associated with long-term mortality and may be used as an independent predictor of long-term outcomes in patients with CAD after PCI.

## Supplementary information

**Additional file 1: Table S1.** Multivariable analysis for CM. **Table S2.** Multivariable analysis for ACM. **Table S3.** Multivariable analysis for MACCE. **Table S4.** Multivariable analysis for MACE.

## Data Availability

Due to confidentiality policies, data will not be shared.

## Abbreviations

ACM: All-cause mortality
CAD: Coronary artery disease
CM: Cardiac mortality
MACCEs: Major adverse cardiovascular and cerebrovascular events
MACE: Major adverse cardiovascular events
PCI: Percutaneous coronary intervention
WAR: White blood cell count-to-apolipoprotein A1 ratio
WBC: White blood cell
