# Supplementary material for: Baseline white blood cell count-to-apolipoprotein A1 ratio as a novel predictor of long-term adverse outcomes in patients who underwent percutaneous coronary intervention: a retrospective cohort study
Source: Lipids Health Dis. 2020 Mar 16;19:43. doi: 10.1186/s12944-020-01206-w (PMC7075035; doi:10.1186/s12944-020-01206-w)
Supplement: Supplementary file 1 — Additional file 1: Table S1. Multivariable analysis for CM. Table S2. Multivariable analysis for ACM. Table S3. Multivariable analysis for MACCE. Table S4. Multivariable analysis for MACE. [file 12944_2020_1206_MOESM1_ESM.docx]

**Table S1. Multivariable analysis for CM.**

| Variables | B | SE | Wald | *P* value | HR (95%CI) |
| --- | --- | --- | --- | --- | --- |
| Age | 0.019 | 0.007 | 7.493 | 0.006 | 1.019(1.005-1.033) |
| Sex | 0.077 | 0.176 | 0.193 | 0.660 | 1.081(0.765-1.526) |
| Smoking | -0.245 | 0.178 | 1.889 | 0.169 | 0.783(0.552-1.110) |
| Drinking | 0.028 | 0.186 | 0.023 | 0.879 | 1.029(0.715-1.481) |
| DBP | 0.000 | 0.006 | 0.001 | 0.981 | 1.000(0.988-1.012) |
| Cr | 0.007 | 0.003 | 6.209 | 0.013 | 1.007(1.001-1.012) |
| UA | 0.000 | 0.001 | 0.003 | 0.959 | 1.000(0.998-1.002) |
| TC | 0.194 | 0.092 | 4.417 | 0.036 | 1.214(1.013-1.454) |
| GLU | -0.022 | 0.024 | 0.867 | 0.352 | 0.978(0.933-1.025) |
| HDL-C | 0.077 | 0.114 | 0.460 | 0.498 | 1.081(0.864-1.352) |
| LDL-C | -0.229 | 0.117 | 3.792 | 0.051 | 0.796(0.632-1.001) |
| WAR | 0.588 | 0.198 | 8.790 | 0.003 | 1.800(1.220-2.655) |
| NLR | 0.204 | 0.149 | 1.871 | 0.171 | 1.227(0.915-1.644) |
| MHR | -0.180 | 0.162 | 1.240 | 0.265 | 0.835(0.608-1.147) |

**Table S2. Multivariable analysis for ACM.**

| Variables | B | SE | Wald | *P* value | HR (95%CI) |
| --- | --- | --- | --- | --- | --- |
| Age | 0.028 | 0.006 | 20.917 | 0.000 | 1.029(1.016-1.041) |
| Sex | 0.086 | 0.157 | 0.304 | 0.581 | 1.090(0.802-1.483) |
| Smoking | -0.096 | 0.157 | 0.369 | 0.544 | 0.909(0.667-1.237) |
| Drinking | -0.044 | 0.166 | 0.070 | 0.791 | 0.957(0.691-1.325) |
| DBP | 0.003 | 0.006 | 0.205 | 0.651 | 1.003(0.992-1.013) |
| Cr | 0.005 | 0.003 | 3.048 | 0.081 | 1.005(0.999-1.010) |
| UA | 0.000 | 0.001 | 0.056 | 0.813 | 1.000(0.999-1.002) |
| TC | 0.132 | 0.086 | 2.396 | 0.122 | 1.142(0.965-1.350) |
| GLU | -0.008 | 0.020 | 0.142 | 0.706 | 0.992(0.954-1.032) |
| HDL-C | 0.090 | 0.099 | 0.824 | 0.364 | 1.094 (0.901-1.330) |
| LDL-C | -0.187 | 0.108 | 3.001 | 0.083 | 0.829(0.671-1.025) |
| WAR | 0.642 | 0.180 | 12.661 | 0.000 | 1.900(1.334-2.705) |
| NLR | 0.327 | 0.136 | 5.781 | 0.016 | 1.387(1.062-1.810) |
| MHR | -0.157 | 0.143 | 1.207 | 0.272 | 0.854(0.645-1.131) |

**Table S3. Multivariable analysis for MACCE.**

| Variables | B | SE | Wald | *P* value | HR (95%CI) |
| --- | --- | --- | --- | --- | --- |
| Age | 0.002 | 0.004 | 0.449 | 0.503 | 1.002 (0.995-1.010) |
| Sex | -0.118 | 0.098 | 1.457 | 0.227 | 0.889(0.733-1.076) |
| Smoking | -0.242 | 0.093 | 6.742 | 0.009 | 0.785(0.654-0.942) |
| Drinking | -0.116 | 0.099 | 1.371 | 0.242 | 0.891(0.734-1.081) |
| DBP | 0.005 | 0.003 | 2.640 | 0.104 | 1.005(0.999-1.012) |
| Cr | 0.001 | 0.002 | 0.099 | 0.753 | 1.001(0.997-1.004) |
| UA | 0.001 | 0.000 | 2.206 | 0.137 | 1.001(1.000-1.002) |
| TC | 0.041 | 0.055 | .553 | 0.457 | 1.042(0.935-1.161) |
| GLU | 0.013 | 0.011 | 1.363 | 0.243 | 1.013(0.991-1.036) |
| HDL-C | -0.010 | 0.074 | 0.020 | 0.889 | 0.990(0.855-1.145) |
| LDL-C | -0.108 | 0.068 | 2.505 | 0.114 | 0.898(0.785-1.026) |
| WAR | 0.438 | 0.103 | 18.121 | 0.000 | 1.549(1.266-1.895) |
| NLR | 0.054 | 0.077 | 0.492 | 0.483 | 1.056(0.907-1.229) |
| MHR | -0.107 | 0.085 | 1.575 | 0.210 | 0.899(0.760-1.062) |

**Table S4. Multivariable analysis for MACE.**

| Variables | B | SE | Wald | *P* value | HR (95%CI) |
| --- | --- | --- | --- | --- | --- |
| Age | 0.001 | 0.004 | 0.026 | 0.873 | 1.001(0.993-1.008) |
| Sex | -0.106 | 0.104 | 1.045 | 0.307 | 0.900(0.734-1.102) |
| Smoking | -0.182 | 0.097 | 3.481 | 0.062 | 0.834(0.689-1.009) |
| Drinking | -0.110 | 0.103 | 1.157 | 0.282 | 0.896(0.732-1.095) |
| DBP | 0.005 | 0.003 | 2.048 | 0.152 | 1.005(0.998-1.012) |
| Cr | 0.000 | 0.002 | 0.028 | 0.867 | 1.000(0.996-1.004) |
| UA | 0.001 | 0.000 | 2.338 | 0.126 | 1.001(1.000-1.002) |
| TC | 0.043 | 0.058 | 0.557 | 0.455 | 1.044(0.932-1.169) |
| GLU | 0.014 | 0.012 | 1.458 | 0.227 | 1.014 (0.991-1.038) |
| HDL-C | -0.026 | 0.081 | 0.102 | 0.749 | 0.975(0.832-1.141) |
| LDL-C | -0.096 | 0.071 | 1.807 | 0.179 | 0.908(0.790-1.045) |
| WAR | 0.432 | 0.108 | 16.002 | 0.000 | 1.540(1.246-1.903) |
| NLR | 0.049 | 0.081 | 0.366 | 0.545 | 1.050(0.896-1.231) |
| MHR | -0.172 | 0.091 | 3.557 | 0.059 | 0.842(0.705-1.007) |
